# Correlation Between Blood Culture Time to Positivity and Vegetation Size in *Staphylococcus aureus* Infective Endocarditis

**DOI:** 10.3390/antibiotics14050456

**Published:** 2025-04-30

**Authors:** Sebastian D. Santos-Patarroyo, Juan A. Quintero-Martinez, Brian D. Lahr, Supavit Chesdachai, Omar Abu Saleh, Hector I. Michelena, Hector R. Villarraga, Daniel C. DeSimone, Larry M. Baddour

**Affiliations:** 1 Division of Public Health, Infectious Diseases and Occupational Medicine, Department of Medicine, Mayo Clinic College of Medicine and Science, Rochester, MN 55905, USA; juanquintero94@hotmail.com (J.A.Q.-M.); chesdachai.supavit@mayo.edu (S.C.); abusaleh.omar@mayo.edu (O.A.S.); desimone.daniel@mayo.edu (D.C.D.); 2 Cardiac Arrhythmia Service, Division of Cardiovascular Medicine, Brigham and Women’s Hospital, Harvard Medical School, Boston, MA 02115, USA; 3 Department of Internal Medicine, Jackson Memorial Hospital, University of Miami Miller School of Medicine, Miami, FL 33136, USA; 4 Division of Clinical Trials and Biostatistics, Mayo Clinic College of Medicine and Science, Rochester, MN 55905, USA; lahr.brian@mayo.edu; 5 Department of Cardiovascular Medicine, Mayo Clinic College of Medicine and Science, Rochester, MN 55905, USA; michelena.hector@mayo.edu (H.I.M.); villarraga.hector@mayo.edu (H.R.V.)

**Keywords:** infective endocarditis, vegetation, length, echocardiography, blood culture, time to positivity, *Staphylococcus aureus*

## Abstract

**Background:** The relationship between vegetation characteristics in *Staphylococcus aureus* infective endocarditis (IE) and blood culture time to positivity (TTP) has not been investigated. This study evaluates the correlation between vegetation length and TTP in patients with *S. aureus* IE. **Methods:** A retrospective cohort study was conducted that included 164 definite cases *S. aureus* IE. Vegetation length was determined by transesophageal echocardiography (TEE), and TTP was measured in hours from the initial time of blood culture incubation to positivity. Correlations between vegetation characteristics and TTP were analyzed using Spearman’s rank correlation coefficient. **Results:** A modest but statistically significant negative correlation was observed between vegetation length and TTP (Spearman ρ = −0.18, *p* = 0.020), suggesting that larger vegetations were associated with shorter TTP. No significant correlations were found for other vegetation characteristics (e.g., vegetation mobility, location, or number) and TTP. **Conclusions:** Larger vegetation size in *S. aureus* IE was associated with shorter TTP. These findings highlight the importance of vegetation size in the pathophysiology of *S. aureus* IE and its role in bacteremia dynamics.

## 1. Introduction

Infective endocarditis (IE) caused by *Staphylococcus aureus* is a life-threatening condition occurring in both community and healthcare settings. The burden of disease is particularly high among individuals with prosthetic heart valves, cardiovascular devices, or a history of injection drug use, and appears to be increasing in these populations [1].

Larger vegetation size, based on echocardiographic measurements, has been linked to worse outcomes in patients with IE. According to guidelines from the European Society of Cardiology (ESC) and the American Heart Association (AHA) [1,2], vegetations ≥ 10 mm in length are associated with an elevated risk of embolic events. Because of this association, guidelines advocate for consideration of early valve surgery to reduce embolic risks and improve patient outcomes.

Blood culture time to positivity (TTP), defined as the interval from sample incubation to detection of pathogen growth, has been identified as an important factor associated with patient outcomes in both bloodstream infections and infective endocarditis (IE) [3]. Shorter TTP has correlated with higher bacterial densities, signifying more severe infections and poorer clinical outcomes in patients with *S. aureus* bacteremia (SAB) [4]. Several studies have demonstrated that a shorter TTP has been associated with increased mortality and higher rates of complications, including IE, thus underscoring its importance as a prognostic marker in SAB [5,6].

Although TTP has been well studied in SAB, its correlation with vegetation characteristics in IE remains unexplored. A 1987 animal model study described the relationship between bacterial concentrations in blood cultures and the number of microorganisms per gram of endocardial vegetation in rats infected with *Staphylococcus epidermidis*. These experimental findings suggest a potential link between TTP as an indirect measure of bacterial concentration and vegetation length [7].

Despite the recognized importance of both vegetation size and TTP, the relationship, if any, between vegetation length and TTP in patients with *S. aureus* IE has not been previously investigated. Therefore, the aim of the current investigation was to determine the degree of correlation between vegetation length and TTP in patients with *S. aureus* IE.

## 2. Results

### 2.1. Clinical Features

The study cohort included 164 patients with a median age of 61.6 years (IQR, 42.1–71.8). The population was predominantly male (62.8%). and White (87.8%). The two most prevalent comorbidities were hypertension (53.7%) and diabetes mellitus (50.0%). Only 13.4% of patients had a history of injection drug use. Native valve IE was the most common form of IE, occurring in 76.2% of the cohort, while prosthetic valve IE was observed in 17.1% and device-related IE in 16.5%. Most IE cases were left-sided (70.1%), with right-sided IE occurring in 28.0%, and bilateral involvement in 1.8% of cases. The median time to positivity (TTP) of blood cultures was 11.0 h, with an IQR of 9.0 to 14.6 h. When TTP was assessed by the side of IE, a TTP of 11.0 h with an IQR of 8.1 to 15.1 h was found in cases of left-sided IE, while a median TTP of 11.0 with an IQR of 9.0 to 14.1 was observed in right-sided IE. No statistically significant difference was found between the groups. Eleven patients (6.7%) received antibiotics before blood cultures were obtained. Surgical intervention for IE was performed in 39 (23.8%) patients (Table 1).

### 2.2. Vegetation Echocardiographic Characteristics

Median designations of echocardiographic findings revealed that the median vegetation length was 10 mm (IQR 7–15), with 57.9% of vegetations measuring ≥ 10 mm. The median vegetation width was 6 mm (IQR 4–10), and the approximated area was 62 mm^2^ (IQR 25–126). The most common location of vegetations was the mitral valve (40.9%), followed by the aortic valve (30.5%) and the tricuspid valve (18.9%). Based on TEE findings, 22.0% of the assessed patients had highly mobile vegetations, and 20.1% of patients had multiple vegetations (Table 2).

### 2.3. Correlation Between TTP and Vegetation Length

**Figure 1:**
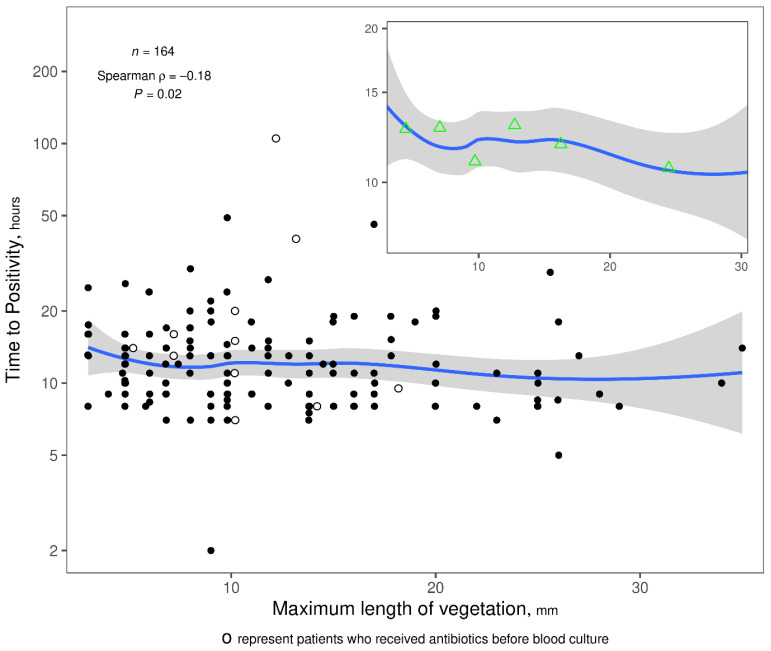
Correlation between the length of a vegetation and blood culture time to positivity. The relationship between vegetation length and TTP was estimated graphically using the loess nonparametric smoother. Each raw data point (TTP vs. length) is one dot in the main plot, and the blue trend line is the loess smoothed curve fit through all the points, with gray bands representing the 95% confidence limits. White dots represents the 11 patients who received antibiotics before blood was drown for TTP calculation. By limiting the y-axis range in the smaller inset plot, the overall decreasing trend is easier to discern. Green triangles represent subgroup estimates obtained by stratifying length into six intervals containing > 20 patients per interval and plotting the geometric mean TTP value against the mean length within the corresponding interval. The geometric mean TTP is not always monotonically decreasing across increasing length intervals.

**Figure 2:**
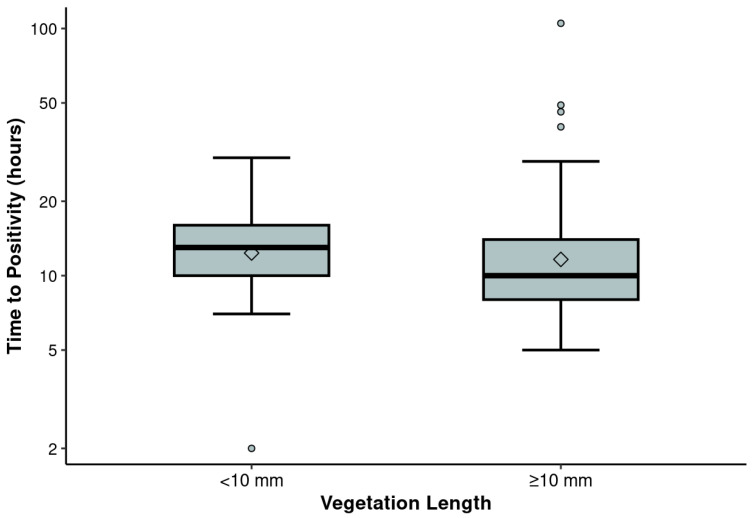
Distribution of time to positivity (TTP) by vegetation length above or below 10 mm. Due to skewness in the distribution, the box plots show the TTP values on logarithmic scale so that the data are more evenly spread and easier to compare between the two groups. Boxes display the interquartile range, with the lines and diamond symbols inside each box representing the median and mean, respectively; vertical lines (i.e., whiskers) extending above and below the box show the range of non-outlier values.

Spearman rank correlation analysis revealed a limited but statistically significant, negative correlation between vegetation length and TTP in blood cultures (Spearman ρ = −0.18, *p* = 0.020). This relationship is estimated in the scatter plot using a loess nonparametric smoother (Figure 1). Further analysis showed consistent findings when vegetation lengths were categorized as either above or below 10 mm. Patients with vegetation lengths < 10 mm (n = 69) had longer TTP values, with a median of 13 h (IQR 10–16), compared to those with lengths ≥ 10 mm (n = 95), who had a median TTP of 10 h (IQR 8–14) (two-sample Wilcoxon test *p* = 0.026). When other vegetation parameters (namely, width, area, number, and mobility) were analyzed, no significant correlations with TTP were identified, and the Spearman ρ values ranged from −0.14 to 0.09. When a cutoff of 10 mm was applied to stratify vegetation length, a comparison between vegetations > 10 mm and <10 mm revealed that larger vegetations were associated with a shorter median (TTP) The Hodges–Lehman estimate of the median difference in TTP between groups with length < 10 vs. ≥10 mm is 1 h (95% CI: 0.00004 to 3) (Figure 2).

A longer vegetation length was independently associated with a shorter time to positivity (*p* = 0.037), even after adjustment for hypothesized confounders including age, vegetation location, native valve endocarditis, intravenous drug use history, and prior antibiotic exposure. In this multivariable model, none of the preselected covariates demonstrated a significant association with time to positivity (Table 3).

## 3. Discussion

To our knowledge, this is the first investigation to analyze the correlation between vegetation length and TTP in patients with IE due to *S. aureus*. Our findings revealed a clinically small but statistically significant inverse relationship between TTP and vegetation length. Other vegetation variables assessed by TEE, including vegetation area, mobility, and multiplicity, were not associated with a shorter time to positivity.

Both vegetation length and TTP are variables that have been associated with negative patient outcomes. Vegetations longer than 10 mm have been linked to an increased risk of embolism and poorer outcomes among patients with IE due to *S. aureus* and other pathogens [8]. Indeed, as previously cited, IE management guidelines have [2,3] highlighted the clinical importance of vegetation size. Moreover, it has been well-established that a shorter TTP, particularly in patients with SAB, has been associated with both a higher risk of underlying IE and mortality [9].

Vegetation length is a strong predictor of embolic events in patients with IE [10,11,12]. The assessment of embolic risk is crucial, and numerous studies have attempted to identify factors that may predict embolism. Comorbidities such as diabetes mellitus and atrial fibrillation have been proposed as predictors, but echocardiographic findings remain the most reliable tool for identifying patients at risk. Although risk scores have been proposed, only IE due to *S. aureus* has been consistently correlated with embolic risk [13]. Integrating existing tools such as echocardiography with blood culture data could be used to create a better predictive model of IE outcomes.

Although the association between larger vegetation size and shorter TTP has not been specifically studied, the correlation between bacterial concentration and vegetation weight was previously explored in a laboratory study published in 1987. In an experimental model of infective endocarditis caused by *Staphylococcus epidermidis*, the investigators demonstrated a relationship between bacterial concentration and the number of microorganisms per gram of endocardial vegetation [7]. This study represents the most comparable finding to our results. While it was conducted in animal models and involved a microorganism with distinct biological characteristics, it provides relevant contextual support. However, differences in microbial profiles and the use of imaging modalities such as TEE in our study limit the direct comparability of the findings.

The prognostic significance of shorter TTP has been evaluated in several studies. Risk scores such as PREDICT I [14], PREDICT II [15], VIRSTA [16], and POSITIVE [5] have been developed to identify SAB patients at low risk for infective endocarditis (IE) who may not require TEE. Among these, only the POSITIVE score incorporated TTP as a criterion for successfully stratifying patients at low risk of IE. Nonetheless, further validation of this scoring system in additional SAB cohorts is still needed.

Two strong variables—TTP and vegetation length—derived from distinct diagnostic modalities (microbiological and echocardiographic) were identified as correlated factors that may help predict outcomes in *S. aureus* IE, a condition associated with a higher complication rate compared to other etiologies [17,18]. Although short TTP has not yet been incorporated into existing risk scores for embolism or other complications in IE, the observed correlation suggests that TTP could serve as an important variable for assessing not only the risk of IE but also the risk of adverse outcomes specifically in *S. aureus* IE cases. Furthermore, the association between shorter TTP and larger vegetations may help guide clinical decision-making regarding early intervention strategies to prevent embolic events in patients with IE.

The current investigation has several limitations. One limitation is the observational study design, which could have introduced confounding factors that were not accounted for in our unadjusted correlation analyses. Also, not all patients who had positive blood cultures were included in the analysis due to TTP not being available by some clinical laboratories. Additionally, the analysis focused solely on *S. aureus* IE, which may limit the generalizability of the findings to that of other pathogens. Among the 164 patients, 11 received antibiotic treatment before blood was drawn for TTP. We assessed the potential impact of this factor on our study outcomes and found no evidence of interference. Consequently, these patients were included in the statistical analysis.

Finally, it is important to note that the small effect detected in our study, as reflected in the measure of correlation between vegetation length and TTP (Spearman ρ = −0.18), suggests a modest association that is statistically, but not necessarily clinically significant.

In conclusion, our findings highlight a potential correlation between vegetation length and blood culture TTP. Subsequent evaluation is needed to confirm this association and determine its clinical utility in predicting *S. aureus* IE outcomes.

## 4. Methods

**Figure 3:**
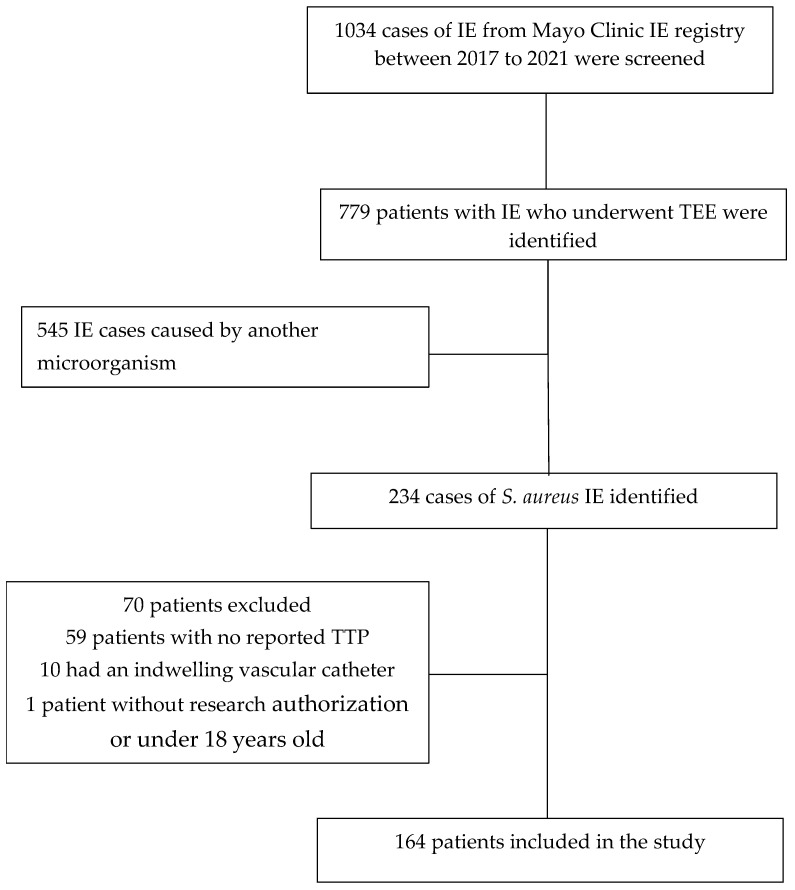
Cohort assembly.

The current investigation is an ancillary study of a previous retrospective cohort analysis of the incidence and risk factors associated with symptomatic embolism among patients with IE [19]. The original study included 779 patients diagnosed with IE according to the modified Duke criteria [20] who were admitted to one of the three Mayo Clinic sites—Rochester, Florida, or Arizona—or to a medical facility within the Mayo Clinic Health System between January 2015 and December 2021. All patients underwent at least one transesophageal echocardiogram (TEE) with a clear description of vegetation(s), thereby making detailed echocardiographic data available for risk factor analysis. For patients with recurrent episodes of IE, only the initial episode was considered. Individuals lacking research authorization, with indwelling vascular catheters or under the age of 18 were excluded. The current study was limited to the subset of patients with IE due to *S. aureus* and excluded the first two years of the original study [19] because blood culture time to positivity (TTP) determinations were not performed during that period. Of the 229 *S. aureus* IE cases seen from January 2017 to December 2021, 164 patients had TTP reported and were used for the current analyses (Figure 3). The study protocol was approved by the institutional review boards of Mayo Clinic and was deemed exempt from requiring informed consent in accordance with 45 CFR 46.116 and HIPAA authorization.

### 4.1. Clinical and Microbiologic Data

Clinical patient characteristics with *S. aureus* IE were abstracted from electronic medical records. Demographic information, comorbidities, risk factors, and microbiological findings were collected. Furthermore, detailed information on vegetation characteristics was extracted from the TEE report during the hospitalization for the IE episode. If multiple TEEs were conducted, then only the first one confirming the presence of a vegetation was included. TTP was automatically recorded, followed by additional testing. Two sets of blood cultures were collected, and the shortest time to positivity (TTP) was selected for analysis, based on the premise that microbial growth could occur in any of the culture bottles. Specifically, the earliest TTP observed between the aerobic and anaerobic bottles was recorded and used for data analysis. For patients who underwent valve surgery, the final TEE performed before surgery was analyzed. The details of TTP processing are described in Comba et al. [21]. Additionally, specific data on the characterization of vegetations was abstracted from each patient’s TEE report during the IE episode of hospitalization.

### 4.2. Echocardiographic Data

As part of the investigation into suspected IE cases, TEE was performed during hospitalization, with data collected based on echocardiographic findings described by expert cardiologists. Level III board-certified echocardiologists conducted a thorough and detailed assessment of the images, adhering to current institutional protocols throughout the hospitalization. In brief, these data included the following vegetation characteristics: length and width (mm) as measured from two-dimensional (2D) TEE, as well as the location, degree of mobility, and number of vegetations. Highly mobile vegetation was defined as echogenic masses that extended beyond the valve coaptation plane, moving with the cardiac cycle. One additional variable derived from these data was approximated area (mm^2^) which was calculated as length multiplied by width. In cases with multiple vegetations, the largest one was used to characterize these features for analysis. If vegetations were present on two or more valves, measurements from each were recorded.

### 4.3. Statistical Analysis

Descriptive statistics are reported as median (IQR [interquartile range]) for continuous variables and percentage (number) of patients for discrete variables. The association between vegetation length and TTP was analyzed using Spearman rank correlation. A graphical depiction of this relationship was created by plotting the TTP data on logarithmic scale against vegetation length and then fitting a smoothed curve to the data using the loess nonparametric smoothing algorithm. Other echocardiographic measurements of the vegetation (area, width, number, mobility) were each analyzed for an association with TTP in the same way. Analyses were performed using R statistical programming (version 4.2.2; R Foundation for Statistical Computing, Vienna, Austria).

## Figures and Tables

**Table 1:** Baseline Clinical Features.

Feature	Overall (N = 164)
Age, years	61.6 (42.1–71.8)
Male gender	62.8% (103)
Race	
White	87.8% (144)
Black/African American	4.9% (8)
American Indian/Alaska Native	1.8% (3)
Other	5.5% (9)
Injection drug use	13.4% (22)
Mitral valve prolapse	4.3% (7)
Bicuspid aortic valve	7.9% (13)
Hypertrophic cardiomyopathy	1.8% (3)
Prosthetic valve	20.1% (33)
Cardiac implantable electronic device	22.0% (36)
Heart failure	44.5% (73)
Chronic kidney disease	40.2% (66)
Myocardial infarction	26.8% (44)
Hypertension	53.7% (88)
Chronic obstructive pulmonary disease	17.1% (28)
HIV	4.3% (7)
Diabetes mellitus	50.0% (82)
Atrial fibrillation	20.7% (34)
Moderate/severe liver disease	9.1% (15)
Metastatic solid tumor	0.0% (0)
Other tumors	16.5% (27)
Charlson Comorbidity Index	3 (1–6)
Definite IE	92.1% (151)
Native valve IE	76.2% (125)
Prosthetic valve IE	17.1% (28)
Device-related IE	16.5% (27)
Surgery for IE	23.8% (39)
Side of IE	
Left	70.1% (115)
Right	28.0% (46)
Bilateral	1.8% (3)
Time to positivity, hours	11.0 (9.0–14.6)
Time to positivity by side of IE	
Left Side IE	11.0 (8.1–15.1)
Right Side IE	11.0 (9.0–14.1)
Antibiotics before blood culture	6.7% (11)

Values represent the median (lower to upper quartile) for continuous variables and percents (frequencies) for discrete variables.

**Table 2:** Echocardiographic vegetation charactheristics.

Feature	Overall (N = 164)
Length of vegetation, mm	10 (7–15)
>10 mm	57.9% (95)
Width of vegetation, mm	6 (4–10)
Area of vegetation, mm^2^	62 (25–126)
Location of vegetation	
Aortic	30.5% (50)
Mitral	40.9% (67)
Tricuspid	18.9% (31)
Right atrium/ventricle	7.9% (13)
Number of vegetations	
Median (IQR)	1 (1–1)
1	79.9% (131)
2	14.0% (33)
3 or more	6.1% (10)
Multiple vegetation	20.1% (33)
Mobility	
None	12.8% (21)
Low	30.5% (50)
Moderate	34.8% (57)
High	22.0% (36)

Values represent the median (lower to upper quartile) for continuous variables and percents (frequencies) for discrete variables.

**Table 3:** Multivariable Model for TTP.

Predictor	β (95% CI)	*p*
Vegetation length †	−0.36 (−0.69, −0.02)	0.037
Age †	−0.02 (−0.54, 0.50)	0.945
Location of vegetation		0.873
Aortic (reference)	0.00	
Mitral	0.17 (−0.49, 0.83)	
Right-Sided	0.15 (−0.66, 0.96)	
IE Type: Native valve	−0.29 (−1.04, 0.46)	0.447
Regurgitation/stenosis for infected valve: Severe	0.32 (−0.42, 1.06)	0.402
IV drug user	−0.22 (−1.19, 0.76)	0.662
Antibiotics before blood culture	0.81 (−0.34, 1.96)	0.166

Abbreviations: β, regression coefficient; CI, confidence interval; IQR, interquartile range; TTP, time to positivity. † Effects of continuous predictors were rescaled using their IQR to estimate the effect of a 1 IQR increase on TTP (i.e., comparing third with first quartiles). Similar regression results were obtained when the model was re-fitted with length treated as a dichotomous variable (≥10 mm vs. 10 mm, β = −0.63; 95% CI −1.20 to −0.06; *p* = 0.031).

## Data Availability

Data available upon request.
